# Health Tract No. 329

**Published:** 1868-09

**Authors:** 


HEALTH TRACT No. 329.

### WRITING.

Many a man's destiny has been made or marred for time and for eternity, by the influence which a single sentiment has made on his mind, by its forming his character for life, making it terribly true, that moments sometimes fix the coloring of our whole subsequent existence. Hence those who write for the public, should do so under a deep sense of responsibility, and endeavor to do it in that healthful and equable state of mind and body, which favors a clear, unexaggerated and logical expression of ideas. If men wrote nothing for print until after forty, the world would be happier and better, for age and a more extensive and accurate observation, cause many a change of sentiment in later life. A clerical Editor and friend published once, that a cup of tea was an admirable aid to the preaching of a sermon. We thought it a dangerous statement; later on, we were called to see him; he had lost his mind! it might just as well have been advised to preach on a glass of brandy. The minister of our college days urged in the pulpit the advantage of saving spare moments, by having a book at hand to read while waiting at the table, or for an expected visitor; he became famed in both continents, but he lost his mind years before he died, at sixty. No one should write when very hungry, or immediately after eating, nor under the influence of any unnatural stimulant, nor while in a passion; else, in this latter case, he will most certainly make a fool of himself. Those who write under a depression of spirits will always write nonsense or, untrue things. Those who write a great deal late at night, will loose their health or die prematurely. The best time for writing with freshness, vigor and logical truthfulness is in the morning, when the brain has been recuperated and renovated by the natural stimulus of healthful sleep, before its force has been expanded or divided on the common affairs of life. No man ought to write over four hours in twenty four, nor over one hour at a sitting; even oftener, it would be better to walk a few minutes, indoor or out, to rest the brain; but always write when the mind takes hold of the subject, when the spirit is on you, be it day or night.

